# Supplementary material for: Variant effect predictor correlation with functional assays is reflective of clinical classification performance
Source: Genome Biol. 2025 Apr 22;26:104. doi: 10.1186/s13059-025-03575-w (PMC12016141; doi:10.1186/s13059-025-03575-w)
Supplement: Supplementary file 1 — Supplementary Material 1. Figs. S1–S6 [file 13059_2025_3575_MOESM1_ESM.docx]

Fig S1


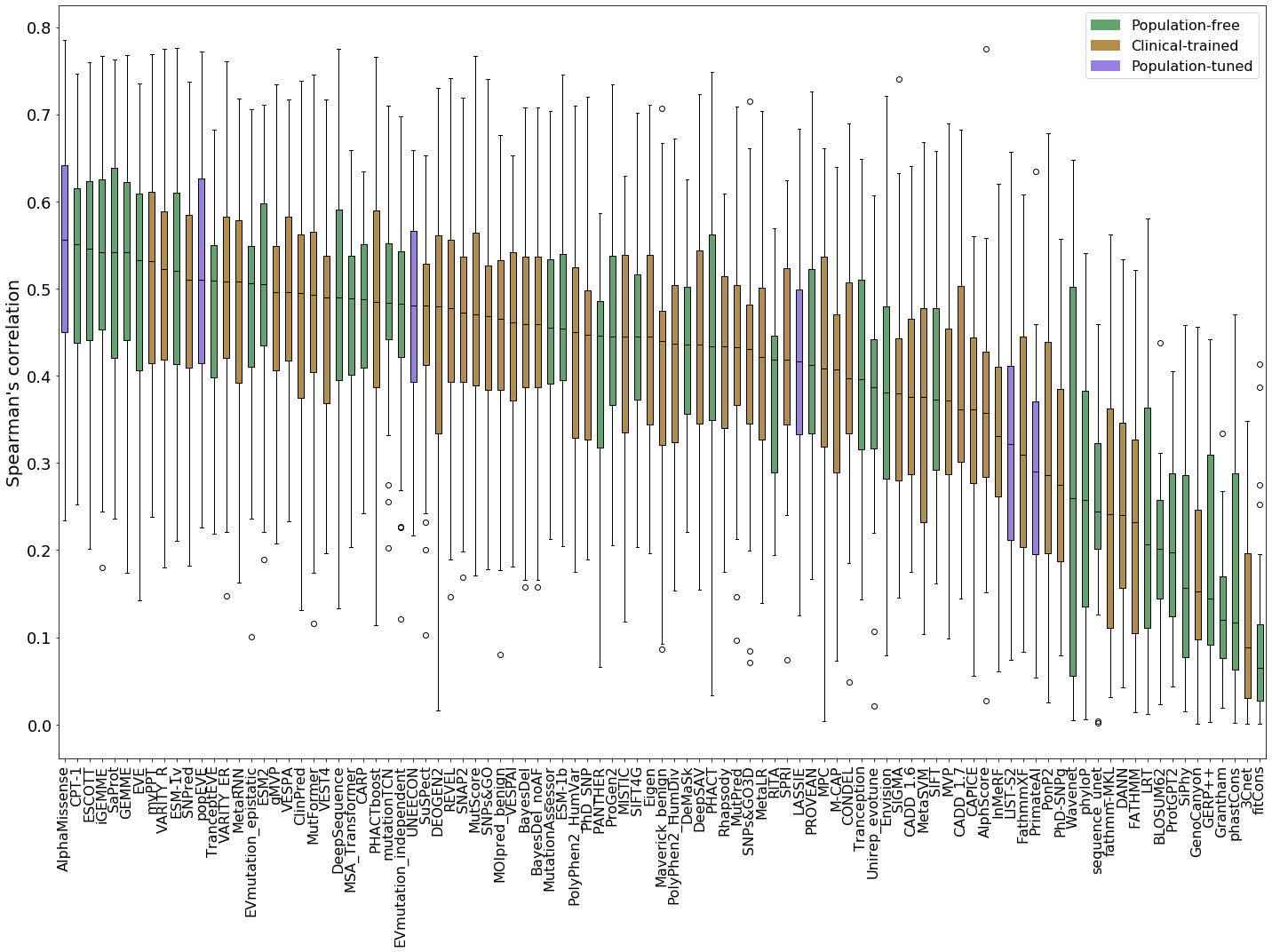


**Fig S1:** **Distribution of Spearman’s correlations of different VEPs across different DMS datasets.** This is effectively the same data presented in Figure 1, but grouped by VEP instead of by protein. Correlations of different VEPs across different DMS datasets. Note that not all VEPs output scores for all DMS datasets, or all variants from individual datasets, so we can not necessarily directly compare correlations in a fair manner, thus motivating the pairwise ranking approach in Figure 2.

Fig S2


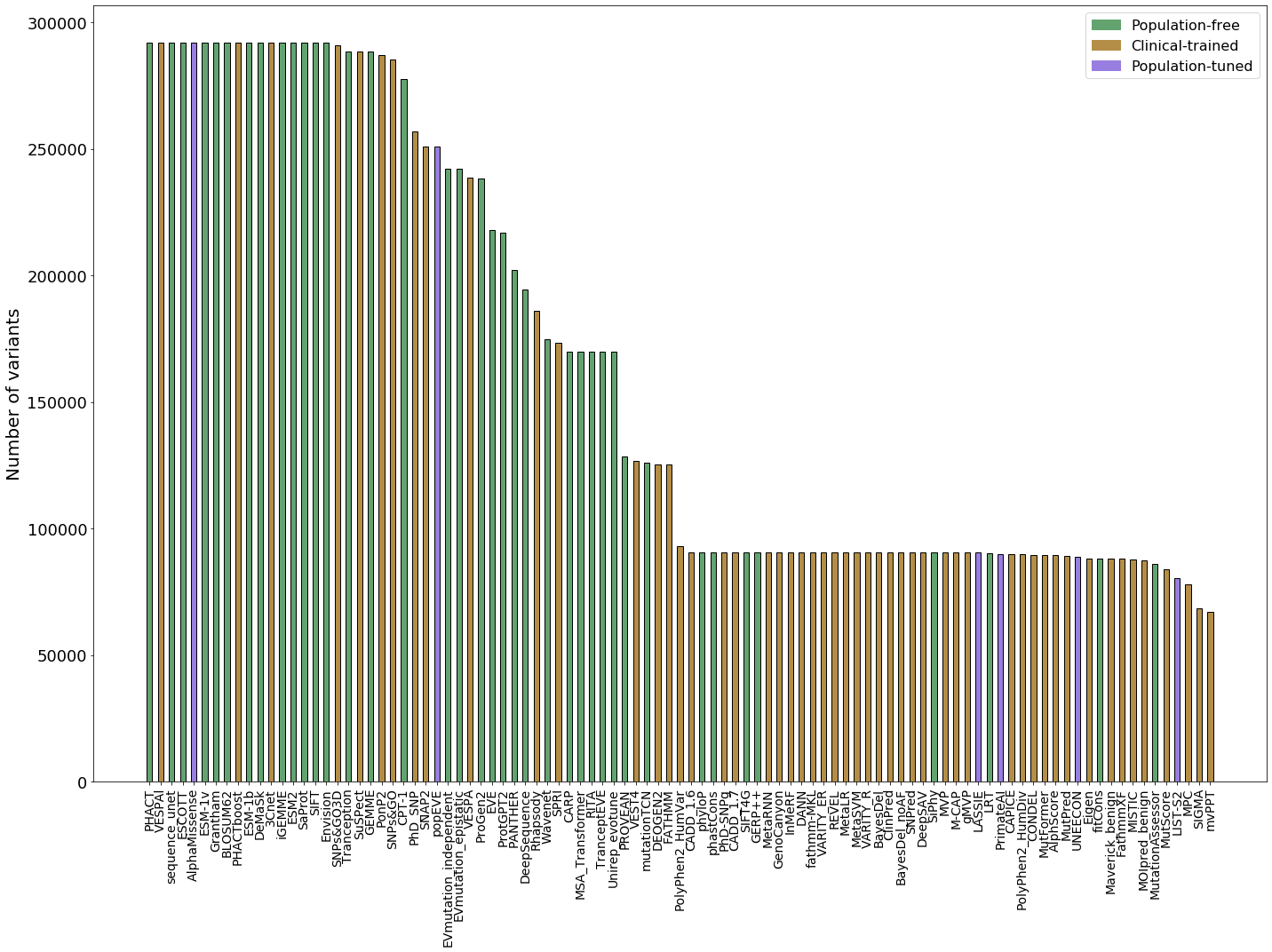


**Fig S2: Variant coverage of amino acid substitutions by VEPs.** The number of variants scored within our dataset by each VEP is shown in this figure.


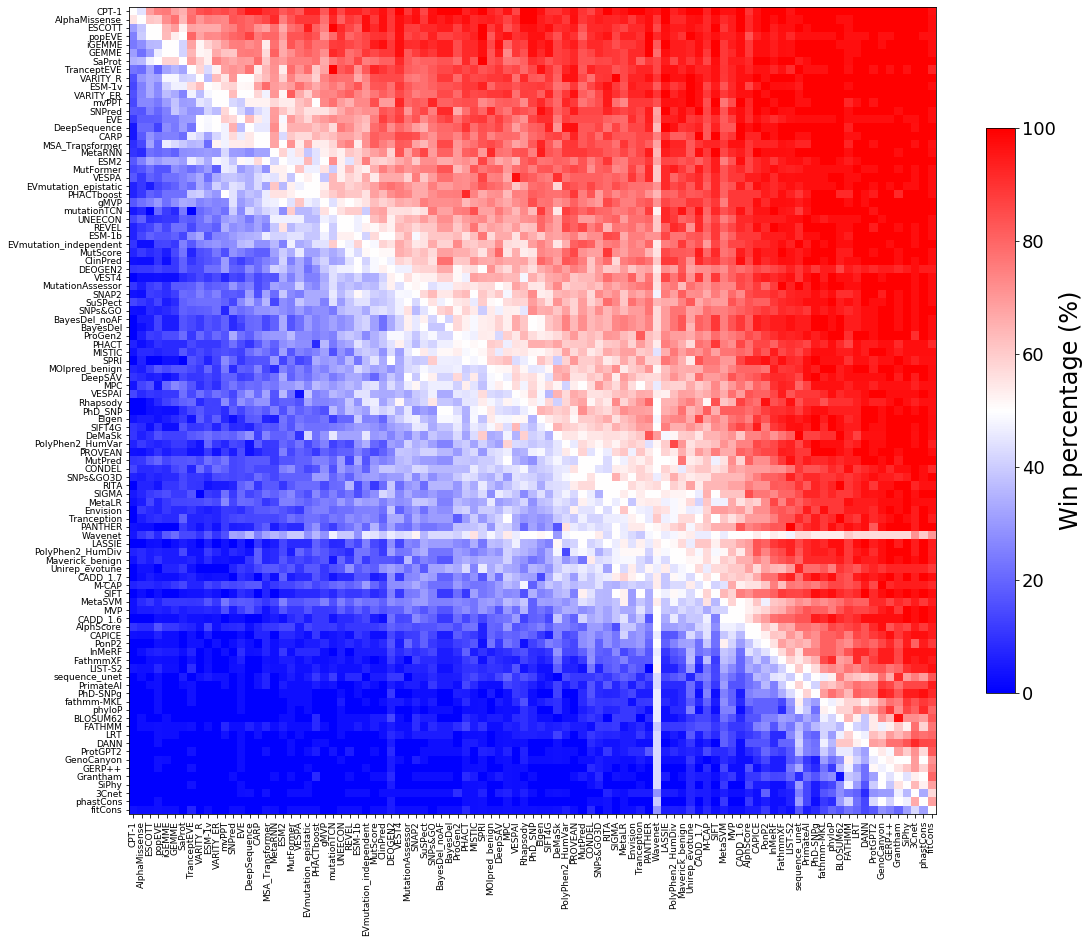
Fig S3

**Fig S3: Heatmap of VEP vs VEP win rates across DMS datasets.** For each pair of VEPs, we compare their Spearman’s correlations with each DMS dataset, considering only variants for which each VEP has a prediction available. The win rate represents the percentage of proteins where the first VEP shows a higher correlation than the second. For example, red values indicate the VEP on the y-axis outperforms the VEP on the x-axis in terms of correlation with DMS measurements across most proteins. The average win rate in Figure 2 represents the mean win rate of each VEP against all other VEPs.

Fig S4


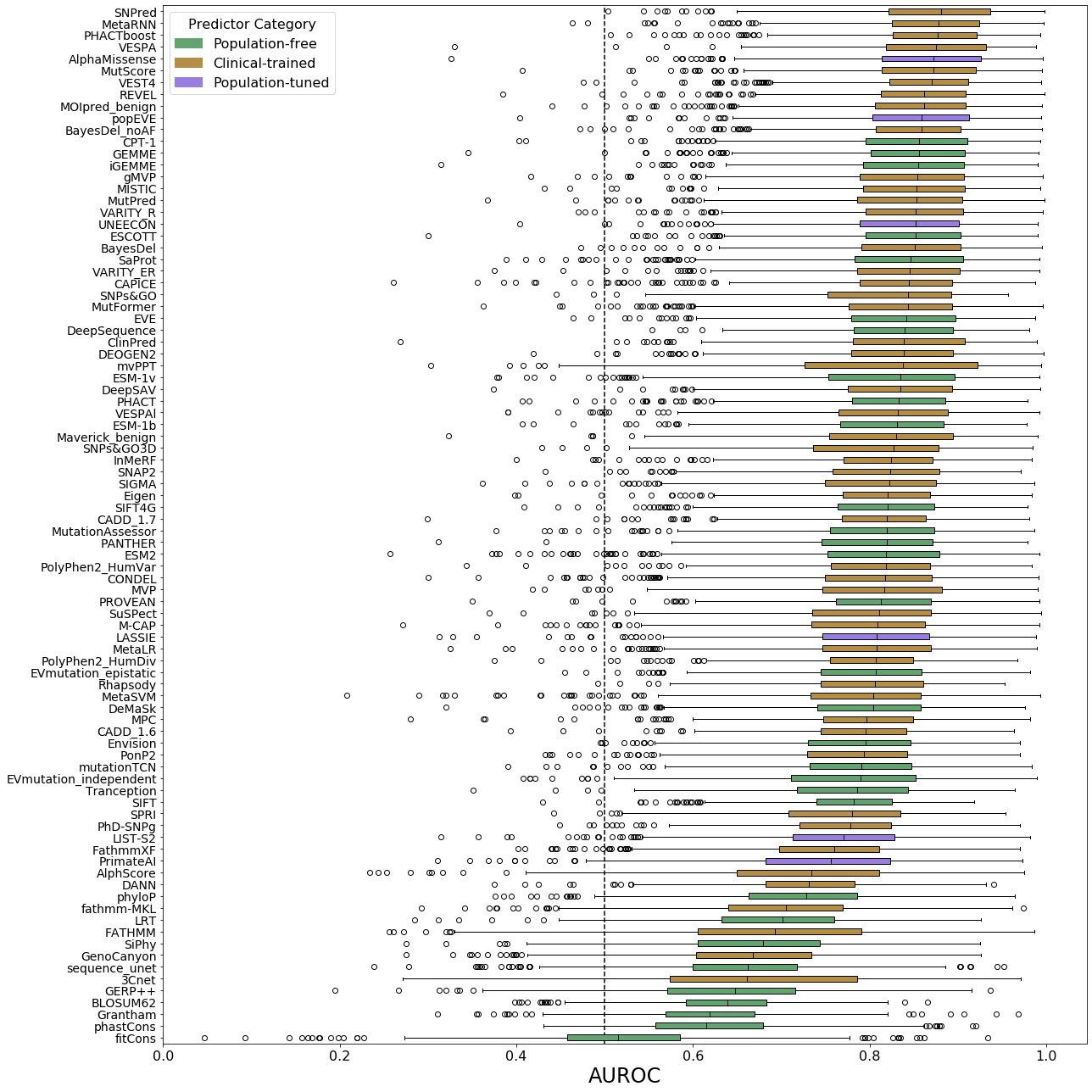
**Fig S4: Distribution of AUROC representing discrimination between pathogenic and putatively benign missense variants for each VEP across different human protein-coding genes.** As for the DMS comparison, not all VEPs output predictions for all proteins or all variants. Thus, the pairwise ranking represents a better reflection of relative performance.

Fig S5


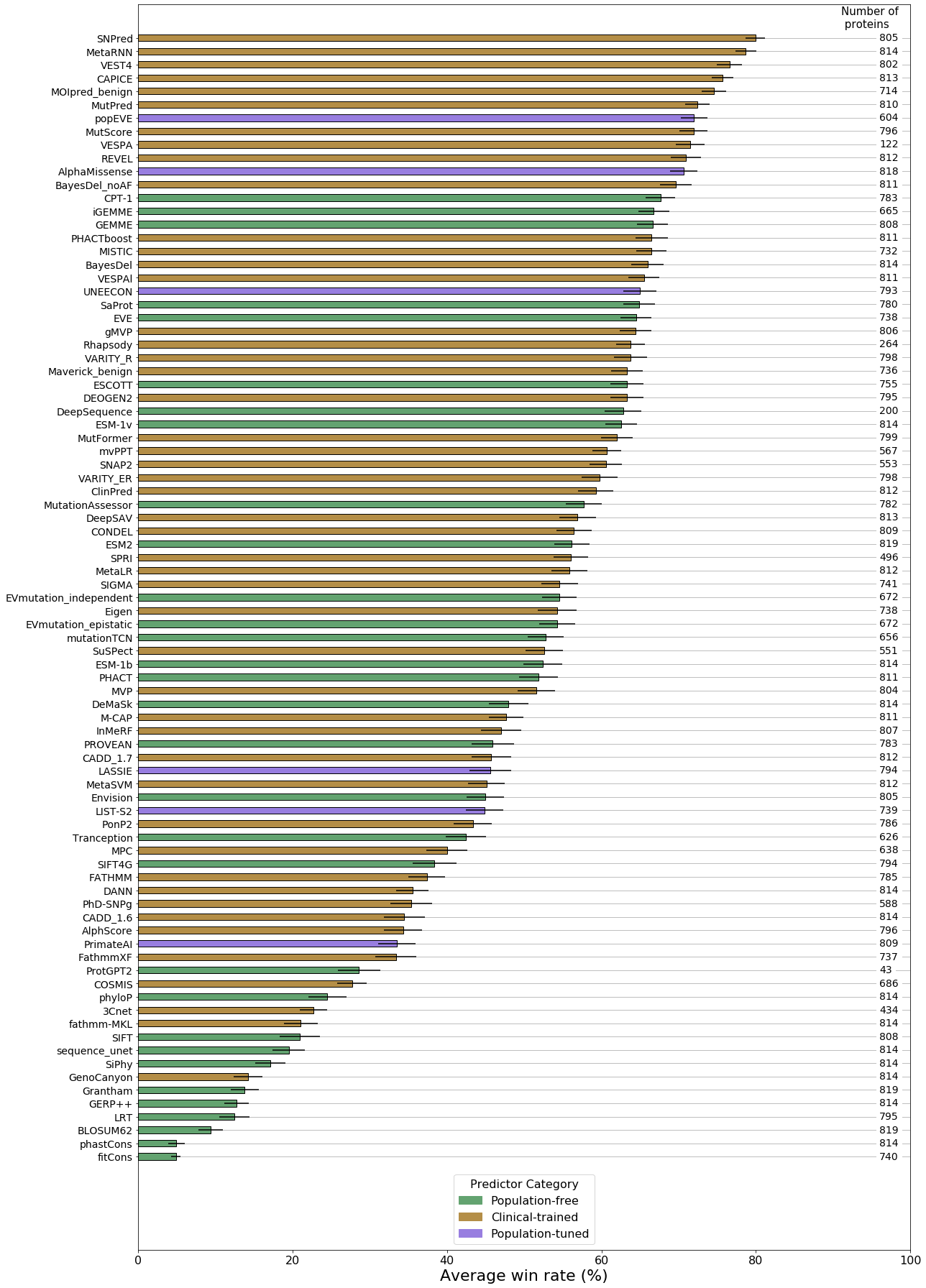


**Fig S5:** **Ranking of VEPs on clinical variant classification using AUPRC.** This utilises the same strategy as in Figure 3, but with precision-recall instead of receiver operating characteristic curves used in pairwise comparisons. Error bars represent the standard error across all comparisons with other VEPs.

Fig S6


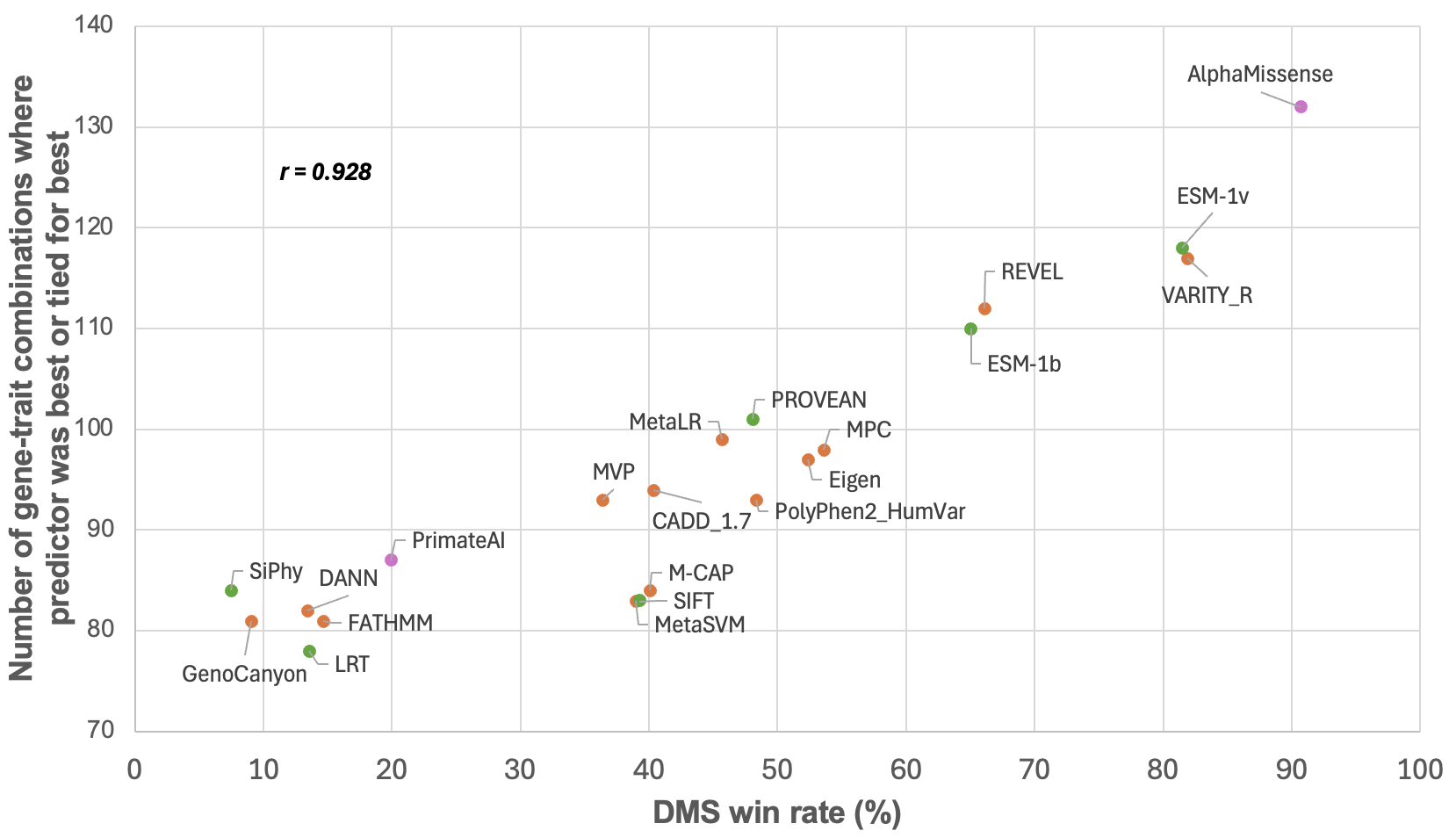


**Fig S6: Correlation between performance against the DMS benchmark from this study and performance in inferring human traits from UK Biobank data from Tabet *et al.*** The number of gene-trait combinations where each predictor was best or tied for best with the top predictor was taken from panel A of Figure 3 in <https://doi.org/10.1186/s13059-024-03314-7>.
